# Endometrial Aspiration Cytology: A Cross-Sectional Comparative Study of Its Efficacy and Sensitivity in Diagnosing Gynecological Disorders

**DOI:** 10.7759/cureus.41699

**Published:** 2023-07-11

**Authors:** Trupti A Dongre, Sabiha A Maimoon

**Affiliations:** 1 Pathology, N.K.P. Salve Institute of Medical Sciences and Research Centre and Lata Mangeshkar Hospital, Nagpur, IND

**Keywords:** endometrial malignancy, endometrial aspiration cytology, gynaecological disorders, cytopathology, endometrium, aspiration cytology, karmann's cannula, exfoliative cytology

## Abstract

Introduction

Endometrial aspiration cytology (EAC) is a noninvasive, rapid, and cost-effective procedure for diagnosing gynecological disorders. This study aimed to validate endometrial aspiration as a routine, safe, and efficient outpatient diagnostic procedure, correlating its findings with histopathology evaluations to facilitate early surgical planning for patients with abnormal uterine bleeding.

Materials and methods

This cross-sectional study involved patients of reproductive, menopausal, and postmenopausal age groups who presented with diverse gynecological concerns that required dilatation and curettage. Endometrial aspiration was performed using a Karman cannula (Angiplast Pvt. Ltd., Vatva, India), and the obtained material was prepared into smears and stained for evaluation. Sampling adequacy, the nature of glandular and stromal cells, phasing of the endometrium, and other abnormalities were assessed and correlated with histology to examine the diagnostic utility of endometrial cytology.

Results

EAC showed 90.66% sampling adequacy with the Karman cannula. The sensitivity of EAC for diagnosing benign and malignant conditions was 88.7% and 100%, respectively. Conditions including secretory endometrium, proliferative phase, tuberculous endometritis, and glandular hyperplasia were diagnosed using EAC and confirmed by histopathology. Six malignancies were successfully diagnosed on cytology smears, while challenges in differentiation and sampling errors were recognized as limitations of the technique.

Conclusions

This study established EAC as a highly sensitive, minimally invasive preliminary diagnostic tool for gynecological disorders, particularly effective in diagnosing malignancy. Despite certain limitations, the procedure’s ease, cost-effectiveness, and safety underscore its potential for routine use by surgeons.

## Introduction

Cervical cytology, commonly known as exfoliative gynecological cytology, is widely used for detecting precancerous and cancerous lesions of the cervix and endometrial and extra-uterine lesions of neoplasia [1]. Abnormal shedding of endometrial cells can be observed in cervical Papanicolaou (Pap) smears or vaginal pool samples, often degenerated and sparse in contrast to the well-preserved and abundant cells seen in direct endometrial samples. While direct endometrial sampling does offer advantages over exfoliative cervicovaginal cytology, due to the greater number of endometrial cells retrieved and fewer degenerative changes, Pap test screening for endometrial carcinoma has not demonstrated a reduction in disease incidence or mortality [2].

Abnormal uterine bleeding (AUB) is a prevalent gynecological issue prompting women to seek medical help. It can be a symptom of various benign and malignant conditions, but in more than half of the cases, it does not have an organic cause and is classified as dysfunctional uterine bleeding [3]. Historically, endometrial biopsy or curettage has been the diagnostic procedure of choice for endometrial lesions, but it is invasive, necessitates hospitalization, and carries risks associated with anesthesia, surgical trauma, and infections [4]. Endometrial aspiration cytology (EAC) has therefore emerged as a viable alternative. This procedure is cost-effective, time-saving, noninvasive, can be conducted on an outpatient basis without the need for anesthesia, and boasts a sensitivity of 80% to 90% and specificity of 90% to 100% [5]. Thus, it has become a preferred option over endometrial biopsy, especially in mass screening programs, reducing the societal burden of diagnosing atypical uterine bleeding in women of all ages.

The aims and objectives of this study were multifaceted. Primarily, we sought to advocate for and establish the use of endometrial cytology as a routine diagnostic modality. We also aimed to validate endometrial cytology as a safe and efficient outpatient procedure capable of providing an adequate cellular yield for a reliable diagnosis. Furthermore, we intended to correlate the findings of EAC with histopathology results.

## Materials and methods

This diagnostic study was conducted over two years (November 2018 to November 2020) in a tertiary care hospital. Patients who presented to the gynecology outpatient department (OPD) with AUB underwent endometrial aspiration and biopsy. The study received approval from the Institutional Ethics Committee (IEC Registration No: ECR/88/Inst/MH/2013, dated April 20, 2013), ensuring that all study procedures adhered to the required ethical standards.

After obtaining a detailed history and conducting a clinical examination, patients underwent endometrial aspiration in the minor operating theater. The aspiration was performed using an endometrial aspiration cannula (Karman cannula, Angiplast Pvt. Ltd., Vatva, India) with a 20-ml syringe under aseptic precautions. Smears prepared from the aspirated material were stained with Hematoxylin and Eosin (H&E) and Pap stains. Slides were examined on the same day for architectural and cytomorphological features, and the results were later compared with histopathology.

In the same session, endometrial curettage was performed by the gynecologist under short general anesthesia following standard procedure. The biopsy was preserved in 10% formalin. Subsequently, sections were stained with H&E, and a pathologist examined histopathology slides. The results were dispatched on the fourth day. Impressions from the cytological analysis were compared with histopathological diagnosis for sensitivity, specificity, and diagnostic accuracy using relevant statistical tests.

Data were analyzed using Stata software Release 10.1 (StataCorp LP, College Station, USA) and OpenEpi software (February 2007 version, Centers for Disease Control and Prevention, Atlanta, USA). The performance characteristics of the cytology test against the gold standard of histopathologic examination (HPE) were estimated as percentages, accompanied by 95% confidence intervals. The kappa statistic was computed to measure agreement and paired data from cytology and HPE were analyzed using McNemar's Chi-square test. A p-value of less than 0.05 was deemed statistically significant.

## Results

The study evaluated 150 cases of gynecological disorders. The average participant age was 51 years, with 32 patients in the perimenopausal group, 100 in the reproductive age group, and 18 in the postmenopausal age group. From the total sample, 14 were reported as inadequate due to insufficient cellularity or the presence of only stromal or endocervical cells. Of the 150 cases included, EAC provided adequate sampling in 90.66% of the instances using Karmann’s cannula. The technique demonstrated a sensitivity of 88.7% for diagnosing benign conditions and 100% for malignant conditions.

Various conditions were diagnosed using EAC and then confirmed via histopathology. These included 35 cases of secretory endometrium, 45 cases of the proliferative phase, one case of tuberculous endometrium, 43 cases of glandular hyperplasia (with 12 showing mild atypia and four cases with atypia), and six malignancies, including five adenocarcinomas and one moderately differentiated endometroid carcinoma. One case involved dysplastic squamous cells. In this instance, it appears that the cannula may not have been correctly maneuvered through the cervical opening, leading to the likely aspiration of only the squamous cells from the cervix. This case was subsequently diagnosed as carcinoma in situ of the ectocervix (Table 1).

**Table 1:** Endometrial cytology diagnosis compared with findings in histopathology Abbreviations: SGH, simple glandular hyperplasia; CGH, complex glandular hyperplasia, MMT, mixed Mullerian tumor.

Cytology Findings	N	Histopathology Findings	N
Secretory endometrium	35	Secretory	28
		Hormone effect	3
		Bleeding endometrium	1
		Proliferative phase	2
		CGH	1
Proliferative Phase	45	Proliferative phase	34
		SGH without atypia	5
		Secretory phase	1
		Secretory hypertrophy	2
		Secretory with e/o bleeding	1
		Inadequate tissue	2
Proliferative with tuberculous endometritis	1	Proliferative with tuberculous endometritis	1
Simple Glandular Hyperplasia	27	Simple Glandular Hyperplasia	15
		SGH with cystic change	6
		Polyp	1
		Bleeding endometrium	1
		Proliferative phase	2
		Hormone effect with mild atypia	1
		Secretory phase	1
SGH with mild atypia	12	SGH with mild atypia	10
		SGH with mild atypia & cystic change	2
SGH with atypia	4	SGH with atypia	4
Bleeding endometrium	2	Secretory with evidence of bleeding	1
		Few fragments of endometrial glands	1
Occasional stromal cells	1	Atrophic endometrium	1
Only few squamous cells with dysplasia	1	Atrophic endometrium	1
Hormone effect	1	Hormone effect	1
Malignancy	5	Adenocarcinoma of endometrium	3
		Adenocarcinoma with MMT-like component	1
		Moderately differentiated endometrioid carcinoma	1
Low grade stromal sarcoma	1	Occasional stromal fragment with dysplastic spindle cells	1
Severely dysplastic squamous cells	1	Carcinoma in situ	1

Upon statistical evaluation, the sensitivity, specificity, and diagnostic accuracy for diagnosing benign endometrium, specifically during the proliferative and secretory phases, were 87.7%, 93.7%, and 92.25%, respectively. Conversely, for diagnosing atrophic endometrium via cytology, the sensitivity was 0%, while specificity and diagnostic accuracy were 100% and 98.5%, respectively. For the diagnosis of hyperplasia and malignancy, sensitivity rates were 88% and 100%, respectively, and specificity rates were 94% and 100%. The diagnostic accuracy of EAC for hyperplasia and malignancy was found to be 92% and 100%, respectively (Table 2).

**Table 2:** Comparison of EAC and HPE diagnostic performance for different endometrial conditions Abbreviations: EAC, endometrial aspiration cytology; HPE, histopathologic evaluation.

Parameter	EAC Cases (n)	HPE Cases (n)	Sensitivity (%)	Specificity (%)	Accuracy (%)
Proliferative	45	39	90.6	93.9	92.9
Secretory	35	28	84.8	93.6	91.6
Hyperplasia	43	37	88	94	92
Atrophic	0	2	0	100	98.5
Malignancy	7	7	100	100	100

**Figure 1:**
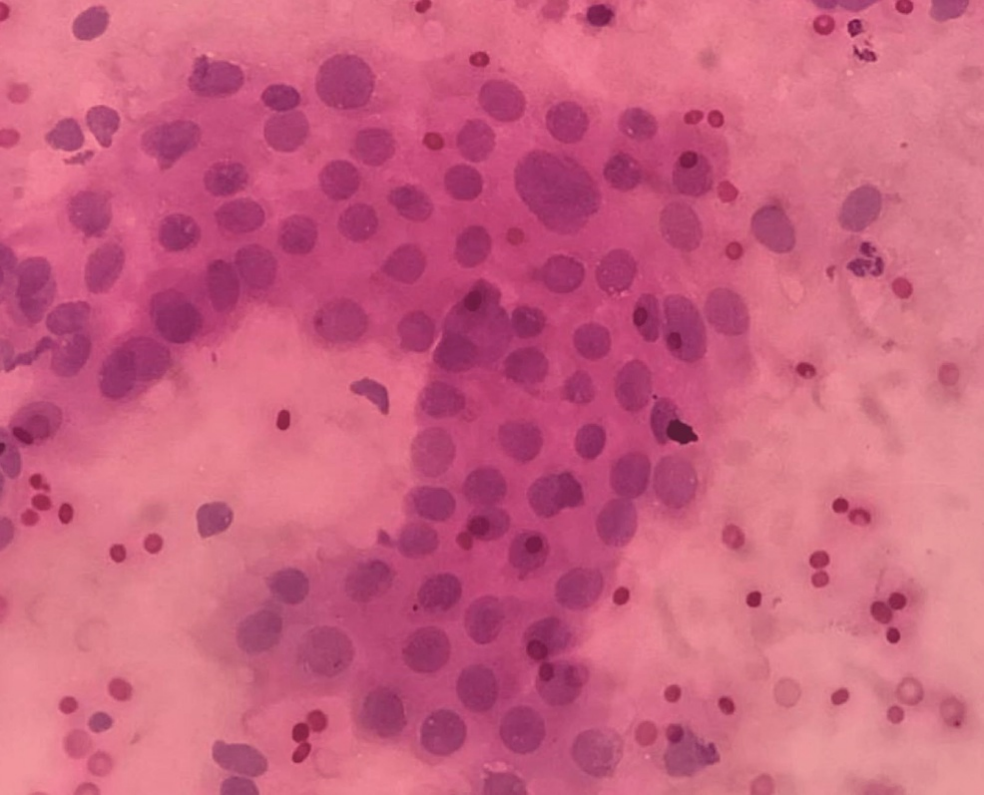
Glandular hyperplasia with atypia (H&E stain, 400x magnification). The image presents mild to moderate anisonucleosis.

**Figure 2:**
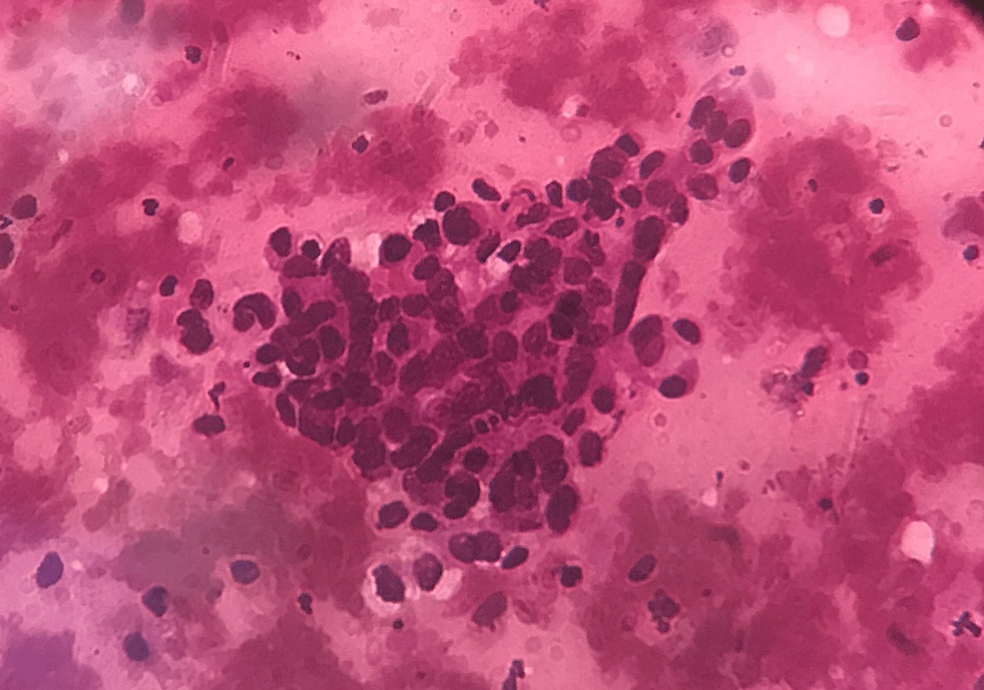
Tuberculous endometritis (H&E stain, 400x magnification). The image shows a cluster of epithelioid cells indicative of granulomatous inflammation.

Cases missed on cytology were due to challenges in differentiation, such as those between the proliferative phase and hyperplasia without atypia and the potential for sampling errors. For example, a polyp was misdiagnosed as glandular hyperplasia with mild atypia (Figure 1), and early secretory changes were missed on two slides. In one case, tuberculous endometritis was diagnosed through aspiration; the patient underwent subsequent treatment, and the diagnosis was confirmed after a hysterectomy (Figure 2). Other misdiagnosis instances were due to improper sample processing, inadequate samples, and sampling errors, which resulted in the inability to diagnose conditions such as bleeding endometrium and hormone effects.

**Figure 3:**
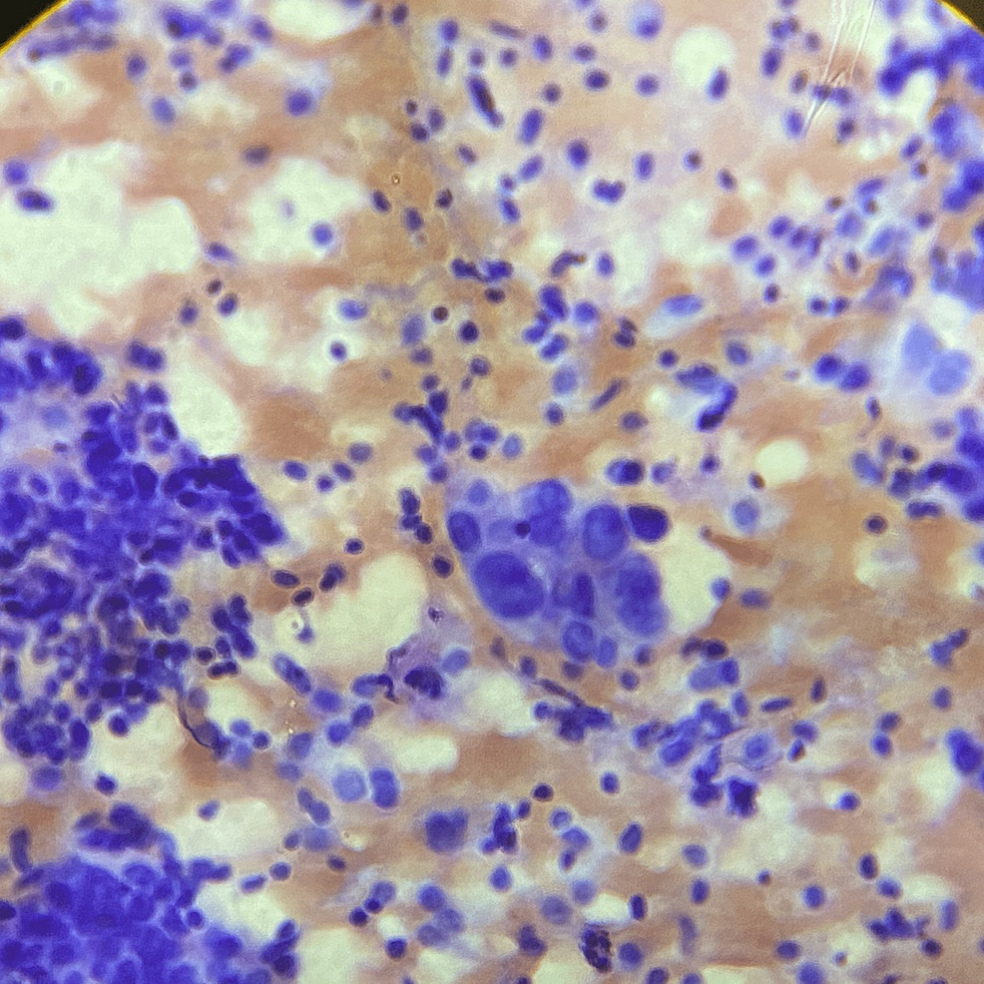
Adenocarcinoma smear (H&E stain, 400x magnification). The image shows scattered tumor cells with marked nuclear pleomorphism and prominent nucleoli.

**Figure 4:**
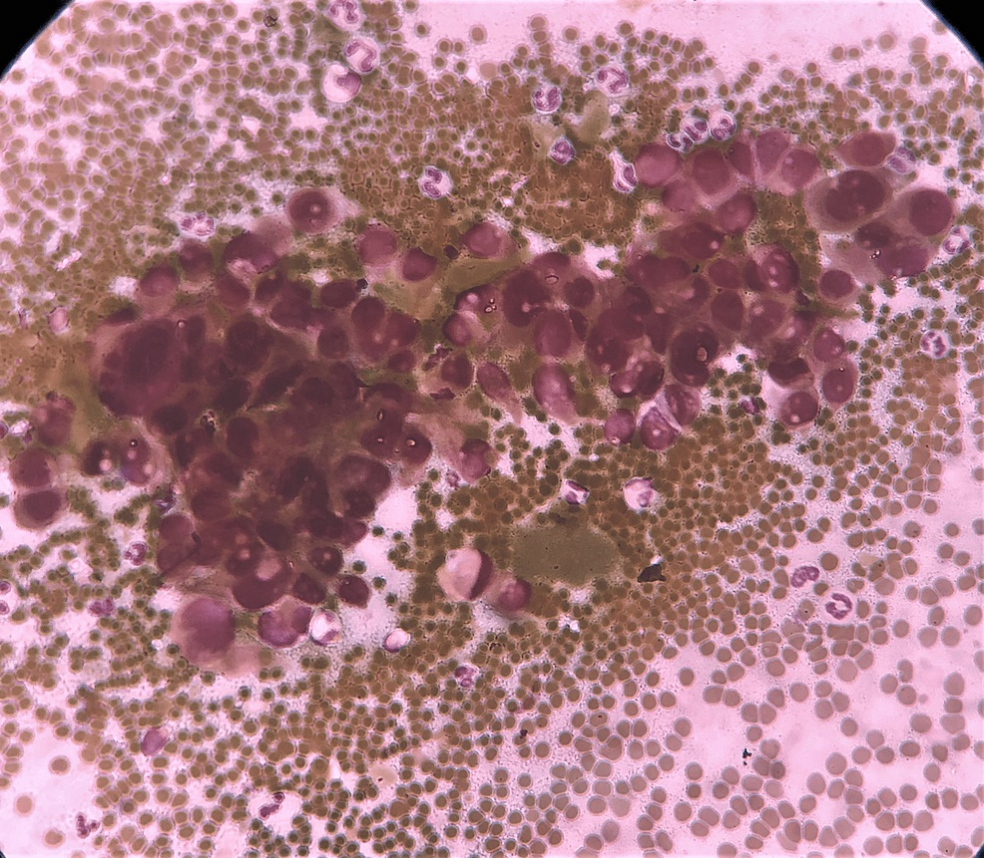
Malignant glandular epithelial cells (Pap stain, 400x magnification). The image depicts scattered glandular epithelial cells showing an increased nucleo-cytoplasmic ratio, hyperchromatic nuclei, anisonucleosis, and pleomorphism.

**Figure 5:**
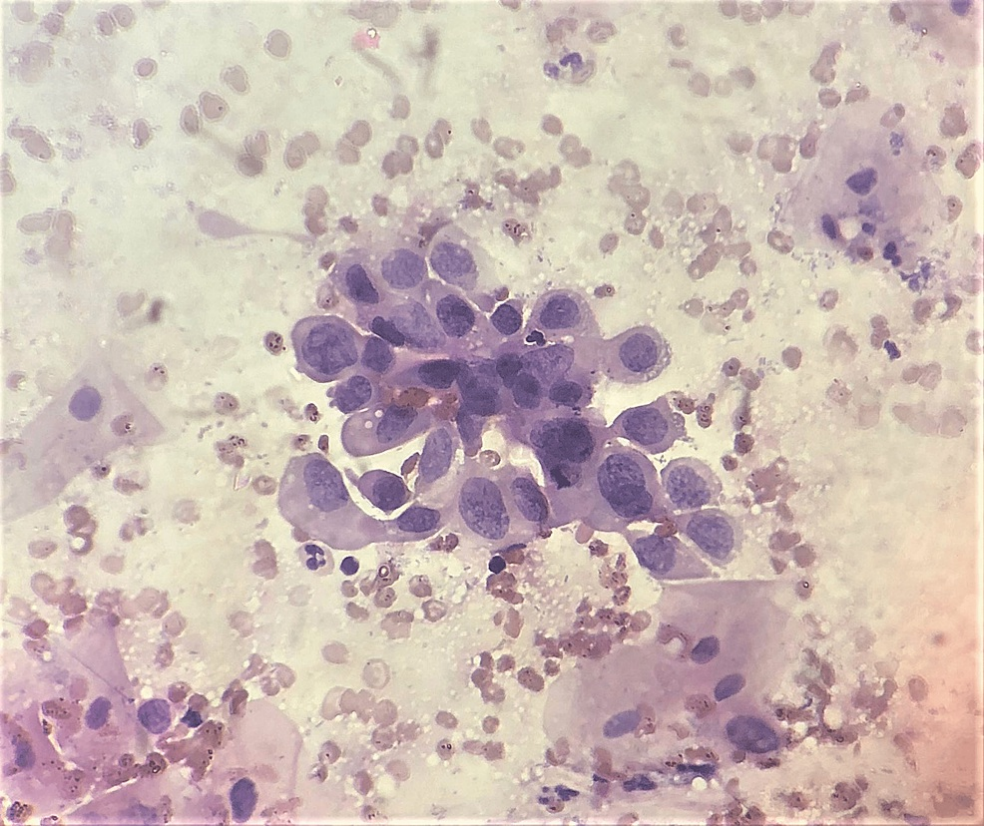
Cytology of dysplastic squamous cells (H&E stain, 400x magnification). The image illustrates highly dysplastic squamous cells.

Despite these challenges, six malignancies were successfully diagnosed on cytology smears, as shown by the characteristics depicted in Figure 3. Notably, the first case of adenocarcinoma required biopsy confirmation due to the indistinct nature of the tumor cells in cytology (Figure 4). Similarly, the second case of a mixed Mullerian tumor, the third case of probable cervical squamous cell carcinoma (Figure 5), and a case of stromal sarcoma were diagnosed through cytology and confirmed by histopathology [6].

## Discussion

Our study underscores the diagnostic utility of EAC in identifying a wide array of gynecological disorders, demonstrating its potential as an efficient, cost-effective, and minimally invasive procedure. The study’s findings align with the well-documented advantages of EAC, such as its speed, cost-effectiveness, and lack of post-procedure adverse effects, enabling early diagnosis and early surgical planning [7,8].

As a first-line screening tool in many settings, EAC is particularly beneficial in atrophic endometrium cases, where obtaining an endometrial biopsy may prove challenging [7,9], although in our study we were unable to obtain sampling from atrophic endometrium. Our study further highlights the ease of creating smears from direct endometrial sampling, a crucial factor contributing to EAC’s viability as an outpatient-based procedure. In contrast, while usable for diagnosing endometrial disorders, cervicovaginal pool samples often provide inadequate samples for diagnosing endometrial pathology.

The high sensitivity of EAC in diagnosing benign and malignant conditions, as demonstrated in our study, aligns with its established capacity to effectively differentiate various endometrial conditions such as secretory endometrium, proliferative phase, tuberculous endometritis, glandular hyperplasia, and malignancies [10-12].

This study confirms the importance of correlating EAC with histopathology for a definitive diagnosis, particularly in instances where architectural patterns are required. While the sensitivity and specificity of EAC in diagnosing malignancies reach 100%, it is less efficient in subclassifying the type of malignancy, a task for which HPE remains the gold standard [7]. As with any medical procedure, EAC is not without its limitations. Despite its high sensitivity in diagnosing gynecological disorders, EAC sometimes struggles to provide an adequate sample for diagnosis, especially in cases with atrophic endometrium [9]. The technique’s inability to consistently provide ample samples for diagnosis underscores the need for additional studies to improve sampling adequacy. The possibility of missing focal lesions limits the utility of EAC as a standalone diagnostic tool [8]. While EAC presents a significant advancement in diagnosing gynecological disorders, its limitations necessitate additional diagnostic modalities for comprehensive evaluation.

Our study had several important limitations. While EAC demonstrated high sensitivity in diagnosing gynecological disorders, it could not always provide an adequate sample for diagnosis, especially in cases with bleeding endometrium, atrophic endometrium, hormone effect, and the early and late phases of the proliferative and secretory cycles. Also, differentiation between certain conditions, such as the proliferative phase, endometrial hyperplasia without atypia, and low-grade adenocarcinoma from complex hyperplasia, proved challenging due to the limited morphological representation in cytology samples, which might not reflect the overall histopathological state of the endometrium. Further, EAC was limited in its ability to diagnose polyps. The possibility of missing focal lesions also limits the utility of EAC. Finally, the study’s findings are based on a single-center experience and may not be generalizable to different settings. Larger, multicenter studies are needed to validate our results further and to explore potential solutions for the challenges encountered in sampling and differentiation.

## Conclusions

This study demonstrated that EAC is a highly sensitive, minimally invasive preliminary diagnostic tool useful in diagnosing gynecological disorders. This straightforward diagnostic tool can eliminate the need for endometrial curettage for benign lesions. In remote areas, patients can access early treatment without significant delay. As a simple OPD procedure without any complications, EAC should be routinely recommended by treating surgeons due to its cost-effectiveness and accurate results.
